# Deep Learning Method on Target Echo Signal Recognition for Obscurant Penetrating Lidar Detection in Degraded Visual Environments

**DOI:** 10.3390/s20123424

**Published:** 2020-06-17

**Authors:** Xujia Liang, Zhonghua Huang, Liping Lu, Zhigang Tao, Bing Yang, Yinlin Li

**Affiliations:** School of Mechatronical Engineering, Beijing Institute of Technology, Beijing 100081, China; 3120180213@bit.edu.cn (X.L.); huangzh@bit.edu.cn (Z.H.); 3120190216@bit.edu.cn (L.L.); 3120180244@bit.edu.cn (Z.T.); 3120190249@bit.edu.cn (B.Y.)

**Keywords:** obscurant penetrating, Lidar, deep learning, 2-D spectrogram image, visual degraded environment (DVE)

## Abstract

With the rapid development of autonomous vehicles and mobile robotics, the desire to advance robust light detection and ranging (Lidar) detection methods for real world applications is increasing. However, this task still suffers in degraded visual environments (DVE), including smoke, dust, fog, and rain, as the aerosols lead to false alarm and dysfunction. Therefore, a novel Lidar target echo signal recognition method, based on a multi-distance measurement and deep learning algorithm is presented in this paper; neither the backscatter suppression nor the denoise functions are required. The 2-D spectrogram images are constructed by using the frequency-distance relation derived from the 1-D echo signals of the Lidar sensor individual cell in the course of approaching target. The characteristics of the target echo signal and noise in the spectrogram images are analyzed and determined; thus, the target recognition criterion is established accordingly. A customized deep learning algorithm is subsequently developed to perform the recognition. The simulation and experimental results demonstrate that the proposed method can significantly improve the Lidar detection performance in DVE.

## 1. Introduction

Light detection and ranging (Lidar) sensors are widely used in detection applications, such as autonomous driving [1,2,3] and unmanned ground vehicles (UGV) [4], due to the characteristics of high resolution [5] and precision [6]. However, when they are applied in degraded visual environments (DVE), such as smoke, fog, dust, and rain, the laser signals of Lidar sensors are attenuated and absorbed by scattering effect and extinction effect of aerosols, thus, it is difficult to extract effective beat signals and detect the targets further [7]. Therefore, using Lidar for target detection in DVE remains a huge challenge [8].

Nowadays, researches on obscurant penetrating mainly focus on fixed distance measurement. Several denoise and backscatter suppression methods have been developed and implemented. Donoho et al. [9] proposed a denoise method, based on the wavelet transformation method. After Huang proposed the Empirical Mode Decomposition (EMD) method, the researchers proposed several EMD-based denoise methods to process the disturbed signal, such as the EMD direct wavelet thresholding method, the EMD interval thresholding method and the EMD-based partial reconstruction [10,11,12,13]. Shamsudin et al. [14] developed a denoise method using SVM and KNN to distinguish the fog and target echo signal in the point cloud data. Heinzler et al. [2] presented a Convolutional Neural Network based (CNN-based) method to distinguish and filter out scatter points caused by adverse weather in point cloud data. Charron et al. [15] proposed a method to remove snow noise by processing point cloud data using a 3-D outlier detection algorithm, which considers the change of point cloud density with the increase of distance from the sensor. In terms of underwater Lidar detection, Illig et al. [16] applied blind signal separation (BSS) to measure and suppress backscattering signal. O’Connor et al. [17] developed several techniques using RF modulation to suppress background clutter and enhance the behavior of target detection, which is quite effective in both forward and backscatter suppression. Djuricic et al. [18] proposed a multiple-pulse laser scanning method to distinguish the target and soft obstacle. However, denoise and backscatter suppression methods are widely analyzed in DVE, yet limited by their computational cost.

Other methods focus on upgrading the optical design of sensors. The OPAL Lidar developed by Neptec company utilize high power emitter with a sensitive receiver to suppress the backscatter from aerosols [19]. Starr et al. [20] fused infrared camera and Lidar for range finding in fire environments. However, the expensive and complex shortcomings limit their application in obscurant penetrating Lidar.

In real application scenarios, Lidar is often installed on moveable platforms, such as unmanned ground vehicles and robots. For practical applications, it is feasible to analyze and apply multiple echo signals when the platform moves. To overcome the above-mentioned deficiencies, this paper presents a novel obscurant penetrating detection method for Lidar, which does not require complex denoising functions. The major contributions of this paper are as follows.

(1) We propose a novel multi-distance measurement method that acquires multiple echo signals in the process of approaching the target. Unlike the fixed-distance measurement, this method acquires multiple echo signals during the movement of the Lidar sensor.
(2) We propose a novel target echo signal recognition method based on 2-D spectrogram images. The 2-D spectrogram images are constructed by using the frequency-distance relation derived from the 1-D echo signals of the Lidar sensor individual cell in the course of approaching target.
(3) We propose a customized deep learning algorithm based on Faster Region Convolutional Neural Network (R-CNN) to recognize the target echo signal and to predict the target distance.

The rest of this paper is organized as follows: Section 2 describes target echo signal recognition method based on 2-D spectrogram images and the deep learning algorithm in detail. To evaluate the effectiveness of the proposed method, the simulation and experimental results are given in Section 3. Section 4 draws the summary and directions for future work of this paper.

## 2. The Obscurant Penetrating Lidar Detection Method

### 2.1. Method Overview

**Figure 1:**
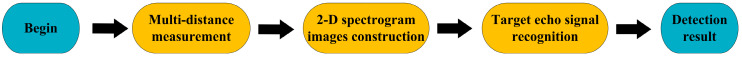
The framework of the obscurant penetrating detection method in this paper.

This paper presents a novel obscurant penetrating detection Lidar method. Among all the obscurants, smoke interference is the most typical interference factor that affects the recognition accuracy of Lidar [21,22]. Thus, this paper is mainly focusing on the smoke environment. The workflow of the method is shown in Figure 1. First, the multi-distance measurement acquires multiple 1-D echo signals during Lidar movement. Next, the 2-D spectrogram images are constructed by using the frequency-distance relation derived from the 1-D echo signals. Then, the deep learning algorithm based on Faster R-CNN extracts the target echo signal based on the joint relationship of the 1-D echo signal sequence. Meanwhile, the target distance will be further predicted. Finally, the Lidar detects the target during the movement.

### 2.2. The Multi-Distance Measurement

**Figure 2:**
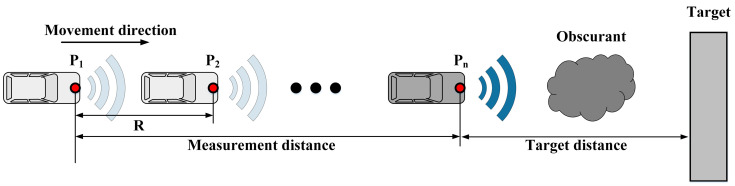
Multi-distance measurement model and application process. The picture shows the multiple measurements made by light detection and ranging (Lidar) during the movement.

First, it is necessary to define the multi-distance measurement. The purpose of explaining the method in detail is to distinguish it from the fixed position measurement method. As Figure 2 illustrates, the Lidar sensor is installed on a moveable platform. In the smoke environment, the Lidar regularly emits signals and receives echoes as the platform moves. *R* is the distance that the platform moves between two probes, *n* is the total number of measurements. Measurement distance is the distance the platform moves between the first detection and the last detection, and the target distance indicates the distance from the target during the last time of measurement. Moreover, the distance between each measurement interval is the same, which can be achieved by controlling the moving speed of the platform.

### 2.3. The 2-D Spectrogram Images

Before proposing the target echo signal recognition criterion based on 2-D spectrogram image, we first analyzed the 1-D echo signals received in different conditions.

**Figure 3:**
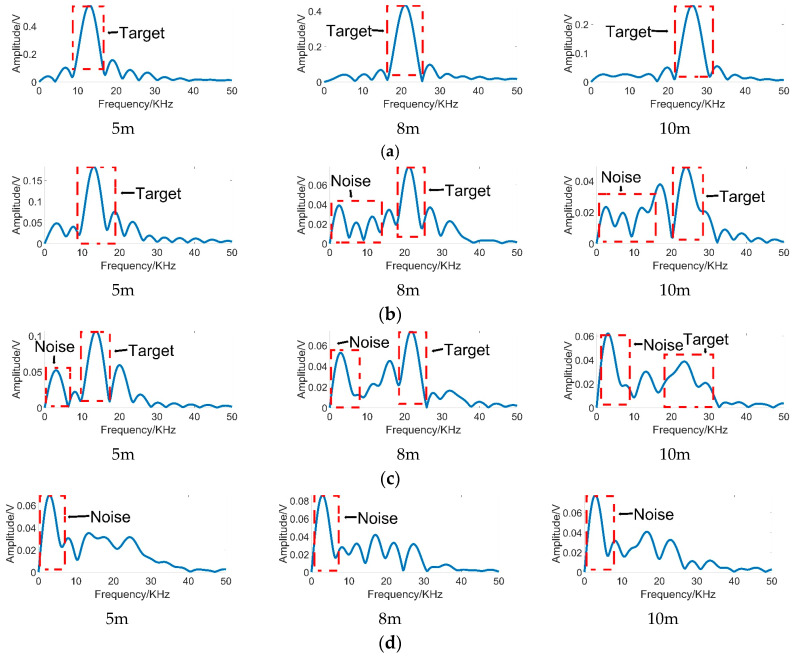
The 1-D echo signals in different conditions. (**a**) In smokeless conditions; (**b**) in conditions of 15 m visibility; (**c**) in conditions of 10 m visibility; (**d**) in conditions of 5 m visibility. The label below each spectrum (‘5 m’, ‘8 m’ and ‘10 m’) indicates the measurement distance from the target.

**Figure 4:**
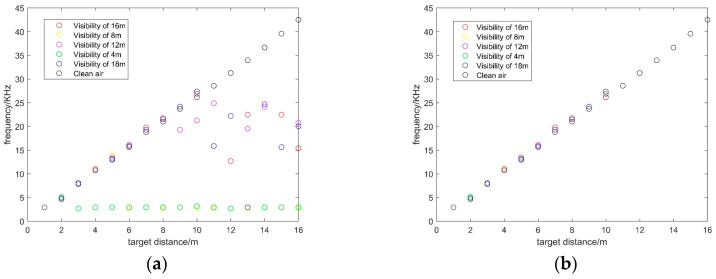
The distribution of beat signal peaks and target echo peaks in different visibility conditions: (**a**) The distribution of beat signal peaks in degraded visual environments (DVE); (**b**) the distribution of target echo signal peaks in DVE.

Figure 3 demonstrates the 1-D echo signals in different visibility conditions. As Figure 3 illustrates, echo signals in Figure 3a are obtained in clear air environments, which can be considered a case of infinite visibility. In the smokeless condition, the highest peak of echo signal demonstrates the location of the target echo signal [7]. It can be found that the frequency of echo signal peak increases with the target distance. Therefore, we visualize the distribution of the frequency of beat signal peak in different visibility conditions as Figure 4a. It can be noticed that, in a smokeless environment, the frequency of target echo signal linearly increases over target distance. Therefore, the relationship between the target distance and the frequency of the target echo signal in smokeless condition can be described as:

(1) $$\begin{array}{c} f=\rho \cdot d \end{array}$$

where ρ is a constant determined by the frequency of beat signal, the period and frequency offset of the FM signal [23,24,25]. However, in the harsh visibility conditions shown in Figure 3, the noise leads to multiple peaks with the rise of smoke concentration. Due to the presence of multiple peaks, it is difficult to extract target echo signal peak in harsh visibility conditions. Moreover, it is not feasible to use Equation (1) to calculate the target distance in a smoke environment.

In addition, it should be noted that, as Figure 3 shows, the noise is mainly distributed in the low frequency part, while the frequency of target echo signal increases over target distance. Thus, it is obvious that the target echo signal is separated from the noise. In order to validate the characteristic, 1-D echo signals in different visibility conditions are analyzed. At first, we design a filter to filter out the peaks of the low frequency part of the echo signals. The filter can be described as:

(2) $$\begin{array}{c} f_{r}=\max\left(0,f-f_{th}\right) \end{array}$$

where $f_{r}$ is the remaining frequency peaks, $f_{th}$ is the threshold value of the frequency. Next, the distribution statistics of peaks in the remaining part are recorded. As Figure 4b shows, the peaks in the remaining part have the same distribution with the peaks in the smokeless environment. It proves that filtering the peak of the low frequency part is helpful to identify the target echo signal. More importantly, the noise is separated from the target echo signal.

**Figure 5:**
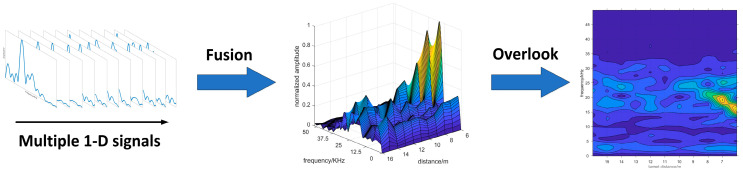
The construction process of 2-D spectrogram image. In 2-D spectrogram image, the yellow part represents the high amplitude value, and the blue part represents the low amplitude value.

Therefore, in order to clearly separate the target echo signal and noise, we began to look for a representation that can fuse signals from multiple distances. Consequently, we proposed the 2-D spectrogram images, which are constructed by using the frequency-distance relation derived from the 1-D echo signals acquired from the multi-distance measurement. The construction process of 2-D spectrogram image is shown in Figure 5. The multiple 1-D signals are combined, as Equation (3) shows. In this process, the multiple 1-D echo signals are normalized in 2-D spectrogram image, which contains the characteristics of the target echo signal and noise. In addition, the size of the 2-D spectrogram image can also be adjusted as needed.

(3) $$\begin{array}{c} A=\frac{1}{M}\left[\begin{array}{c} P_{1}\\ P_{2}\\ \begin{array}{c} \vdots \\ P_{n} \end{array} \end{array}\right] \end{array}$$

where *A* represents the matrix of the 2-D spectrogram, $P_{i}$ represents the sequence of the *i*-th 1-D echo signal, *M* is the maximum value of all 1-D echo signals.

**Figure 6:**
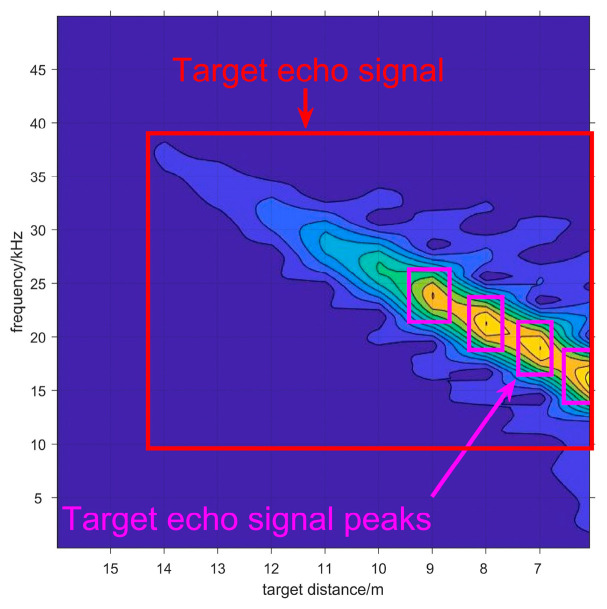
The 2-D spectrogram image in smokeless environment. The target echo signal and target echo signal peaks are annotated in the figure.

The most significant advantage of 2-D spectrogram image is that it demonstrates the amplitude–frequency characteristics of target echo signal during the process of approaching the target, as the noise is separated from the target echo signal. Therefore, the target echo signal can be demonstrated as Figure 6, as the correlation of adjacent data in the sequence is very obvious. Moreover, in order to further measure the target distance, the positions of target echo signal peaks are labeled as well. When the target echo signal is recognized in the spectrogram image, it indicates that the target is detected.

### 2.4. The Target Echo Signal Recognition Algorithm

**Figure 7:**
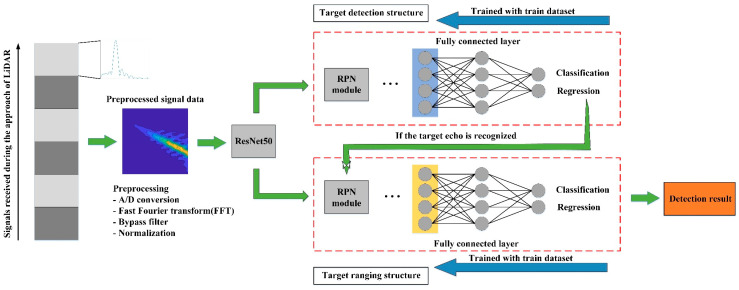
The structure of the proposed algorithm.

The target echo signal can be annotated in the 2-D spectrogram image, while the target frequency linearly increases over target distance. Therefore, a novel signal recognition algorithm based on the visual signature extracted from the echo signal’s time-frequency representation is presented for target detection. As Figure 7 illustrates, two Faster R-CNN networks are utilized in the algorithm, one for signal recognition, and the other is used for target ranging further.

**Figure 8:**
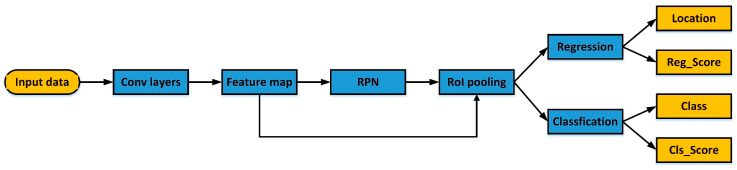
The Faster R-CNN workflow diagram.

Faster R-CNN contains two modules: the region proposal network (RPN) module, and the Fast R-CNN detection module [26]. The RPN module is utilized for extracting candidate regions. Then, the Fast R-CNN module classifies the extracted candidate regions into target and background. As Figure 8 shows, the Faster R-CNN consists of two full-connection layers: a regression layer and a classification layer. The former refines and corrects the proposal regions, so the predicted bounding box is closer to the ground truth. The latter classifies the proposal regions.

**Figure 9:**
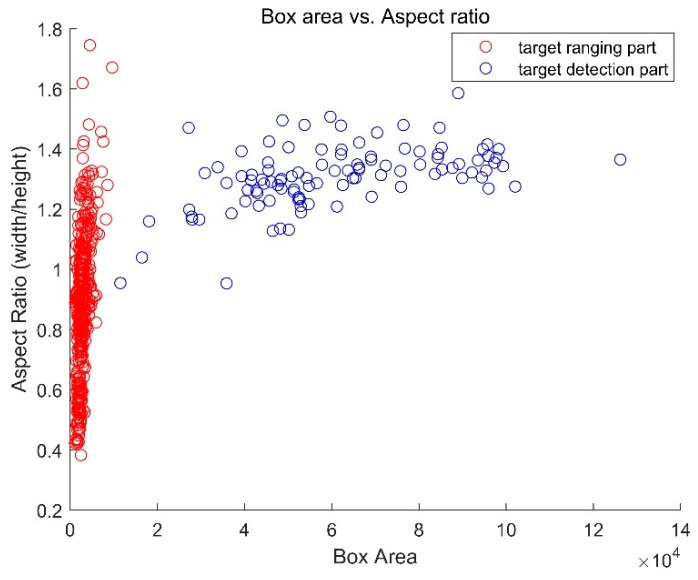
Illustration of the distribution of the two anchors according to the box area and the aspect ratio.

In RPN, the proposal regions are predicted based on the preset anchors’ scales. Two types of anchors are applied for the detection network and the ranging network respectively. The inputs’ scales are clustered to optimize the parameters. As is shown in Figure 9, due to the huge difference between the two types of anchors, two RPN modules are adopted, to predict the proposal regions respectively.

We now describe the loss function applied for training the algorithm. As analyzed from above, the loss function is divided into detection part and ranging part. The first output $L_{reg\_det}\left(t_{i},t_{i}^{g}\right)$ determines the target’s coordinate, where $t_{i}$ is a vector and contains $(t_{x}$, $t_{y}$, $t_{w}$, $t_{h})$, representing the predicted offset of the anchor’s location, and $t_{i}^{g}$ represents the true offset of the anchor relative to the ground truth. The second output is a cross-entropy loss function of binary classification, denoted as $L_{class\_det}\left(p_{i},p_{i}^{g}\right)$, where $p_{i}$ estimate the presence of target in the foreground, and $p_{i}^{g}$ represents the ground truth [26]. The last two of the four loss functions, $L_{reg\_rag}\left(t_{j},t_{j}^{g}\right)$ and $L_{class\_rag}\left(p_{i},p_{i}^{g}\right)$ evaluate the ranging part of the algorithm. The descriptions of these losses are shown as:

(4) $$\{\begin{array}{c} \begin{array}{c} L_{reg\_det}=\lambda \frac{1}{N_{reg\_det}}\underset{i}{\sum }p_{i}^{*}R\left(t_{i}-t_{i}^{*}\right) \end{array}\\ L_{class\_det}=\frac{1}{N_{class\_det}}\underset{i}{\sum }-\log\left[p_{i}p_{i}^{*}+\left(1-p_{i}\right)\left(1-p_{i}^{*}\right)\right]\\ L_{reg\_rag}=\lambda \frac{1}{M_{reg\_rag}}\underset{i}{\sum }p_{i}^{*}R\left(t_{i}-t_{i}^{*}\right)\\ L_{class\_rag}=\frac{1}{M_{class\_rag}}\underset{i}{\sum }-\log\left[p_{i}p_{i}^{*}+\left(1-p_{i}\right)\left(1-p_{i}^{*}\right)\right]\\ L=L_{reg\_det}+L_{class\_det}+L_{reg\_rag}+L_{class\_rag} \end{array}$$

where $p_{i}$ and $t_{i}$ are the prediction probability and predicted coordinate, $p_{i}^{*}$ and $t_{i}^{*}$ are the label and coordinate of ground truth, λ is weight balance parameter, we set it to 10, R is the Smooth L1 function, $N_{reg\_det}$ and $M_{reg\_rag}$ are the number of anchors in the two networks respectively, $N_{class\_det}$ and $M_{class\_rag}$ are the number of mini batch sizes in the two networks, respectively [26]. The sum of the above four losses is minimized until convergence, indicating that the network has finished training.

So far, the recognition algorithm has been proposed, the target distance can be calculated by the location of target echo signal peak as:

(5) $$\begin{array}{c} D=\frac{\left(n-1\right)R}{\sum_{i=2}^{n}(f_{i}-f_{i-1})}f_{n} \end{array}$$

where *D* is the predicted distance of the Lidar from the target, $f_{i}$ is the frequency of target echo signal peak, *n* is the total number of measurements, R is the distance between each measurement.

Average precision (AP) and average ranging error (ARE) are applied as evaluation indicators. AP evaluates the performance of the detection part and ARE evaluates ranging part. AP evaluates the detection performances under different threshold conditions. The higher the AP value, the better the detector’s detection performance. Meanwhile, the ARE is given as:

(6) $$\begin{array}{c} ARE=\frac{1}{N}\overset{N}{\underset{0}{\sum }}\left|D-R\right| \end{array}$$

where D is predicted distance calculated by Equation (5), R is the actual distance between the detector and the target. The lower the ARE, the better the detector’s ranging performance.

## 3. Simulation and Experimental Results

In order to more realistically analyze the detection performance of the proposed method, the recognition algorithm was evaluated by simulation and experimental echo signals, respectively. Subsequently, the evaluate results were then compared to the other algorithms under the same conditions. In the end, the evaluate results in different visibility conditions were analyzed.

### 3.1. The Simulation Results

#### 3.1.1. The Simulation and Training Settings

**Figure 10:**
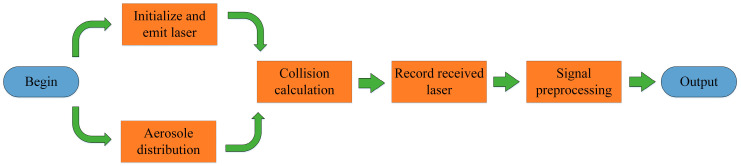
The simulation setup process of echo signal in obscurant environments.

The entire simulation process is shown in Figure 10. The Monte Carlo model was adopted to simulate the echo signals of Lidar in DVE [27,28,29]. The delay time and amplitude of the received signal can be calculated by calculating the time when the photon enters the detector and the energy of all the photons received at that time. The beat signal is simulated after mixing with the local oscillator signal and low-pass filtering. In order to simulate the real scene, the simulation can change the scattering coefficient and extinction coefficient of smoke aerosol, according to the different smoke concentrations. The relationship of visibility and smoke concentration can be expressed as:

(7) $$\begin{array}{c} v=\sqrt[{y}]{\frac{c_{0}}{M_{c}}} \end{array}$$

where the $M_{c}$ is the smoke concentration, v is the visibility, $c_{0}$ and y are constants, depending on the aerosol type, and in a smoke environment, the values of these two constants are 37.3 and 1.07 respectively [30].

**Figure 11:**
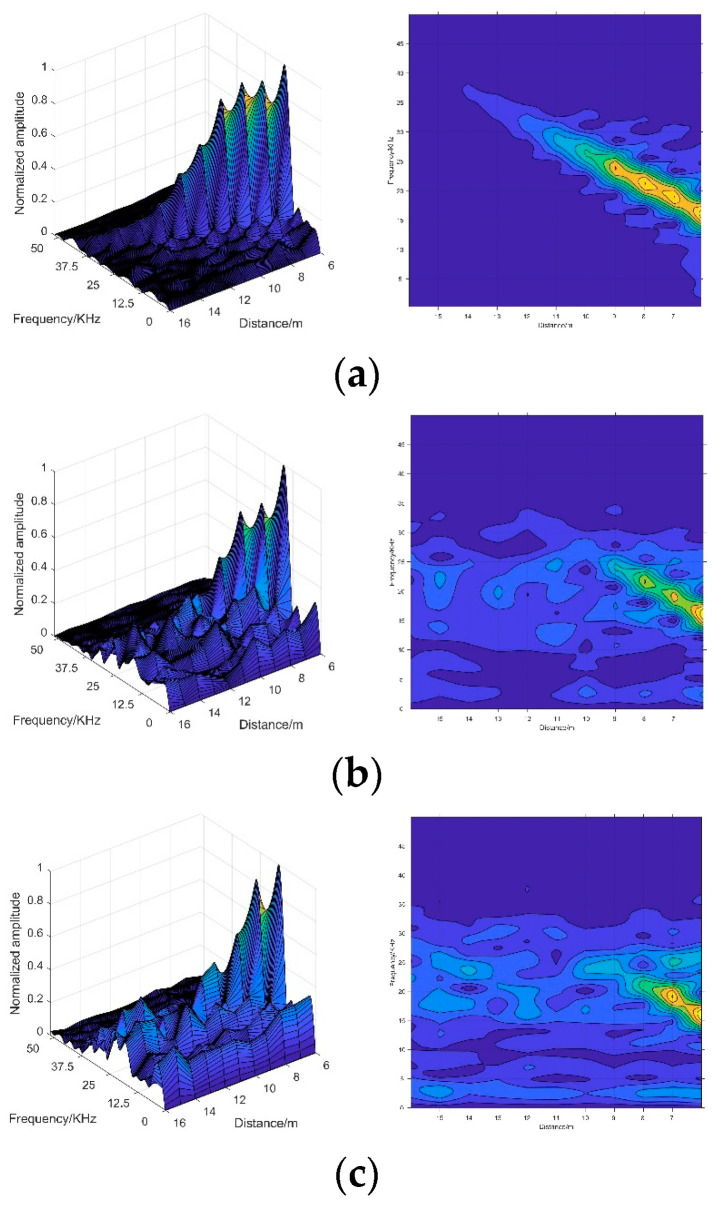

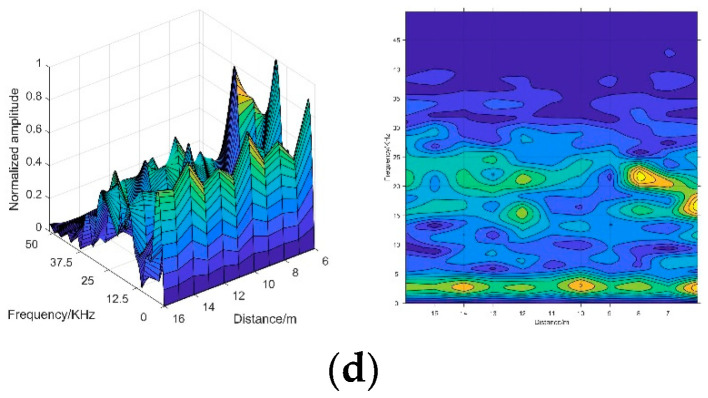
The 2-D spectrogram images of different visibility conditions: (**a**) spectrogram image in smokeless condition; (**b**) spectrogram image in a condition of 15 m visibility; (**c**) spectrogram image in a condition of 10 m visibility; (**d**) spectrogram image in a condition of 8 m visibility.

A total of 135 2-D spectrogram images were simulated in different visibility conditions, ranging from 1 m to 15 m, and the training set and test set were separated as 7:3. Figure 11 shows the 2-D spectrogram images constructed from the simulated signals. The construction detail of the 2-D spectrogram image is shown in Table 1.

Afterwards, we annotated the target echo signals. As Figure 3 shows, the amplitude of target echo signal becomes smaller and smaller as the smoke density increases. In conditions with 1 m, 2 m, 3 m visibility, the target echo signals were too weak to annotate. In order to further evaluate the extreme performance of the proposed algorithm, we shortened the measurement distance in harsh visibility conditions. Measurement times refers to the number of measurements during the multi-distance measurement.

Then, the stochastic gradient method (SGD) was applied in the optimization process, of which the number of iterations was 80,000, the momentum and the weight decay were 0.9 and 0.0005, respectively. The initial learning rate of the first 60,000 mini-batch was 0.0001, and the last 20,000 mini-batch was 0.00001. Since ResNet50 has a better detection performance than VGG16, we applied ResNet50 as the pre-trained module in the algorithm. In total, the algorithm was trained for 100 epochs from scratch. We applied NVIDIA’s GTX 960M graphics card for accelerating computing.

#### 3.1.2. Detection Results of the Simulated Signals

(1) Comparison with Other Deep Learning Algorithms

The deep learning detection methods are divided into region proposal based and non-region proposal based algorithms. The representative of the former is R-CNN, Fast R-CNN, and Faster R-CNN, the representative of the latter is You Only Looks Once (YOLO). The region proposal based algorithm has a higher recognition accuracy, while the non-region proposal based method, such as YOLO, has better real-time performance with lower detection accuracy. Moreover, YOLO cannot detect the small target in the image [31]. We investigated the performances of different algorithms (YOLO, R-CNN, Fast R-CNN, and Faster R-CNN) and different resolutions (227 × 227 and 500 × 500).

**Figure 12:**
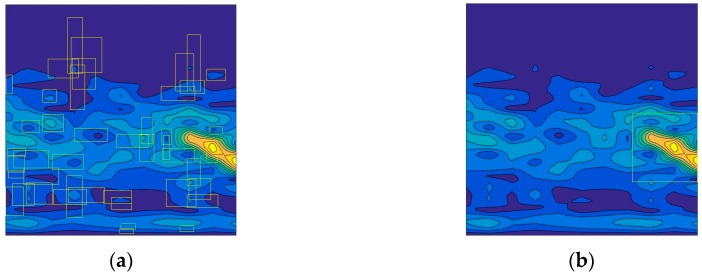
The detection results by selective search and the region proposal network (RPN): (**a**) results of selective search; (**b**) target echo signal location by RPN.

As can be seen from Table 2, YOLO cannot effectively identify the target echo signal in the 2-D spectrogram image, but, compared with other algorithms, it takes the fastest time, which is consistent with the previous view that the non-region proposal based method cannot identify small targets. Therefore, we focus on comparing the recognition results, based on the region proposal based algorithms. The performances of models with 500 × 500 inputs are better than those with 227 × 227, as larger inputs contain more detail. At the same time, the larger the input image, the more time it consumes. By using 500 × 500 instead of 227 × 227, the AP of Faster R-CNN has been improved by about 6.4%, and the ARE has been reduced by 1.67 m. In 500 × 500 conditions, the AP of R-CNN and Fast R-CNN are 75.1% and 86.3% respectively, lower than that of Faster R-CNN. While R-CNN and Fast R-CNN adopt selective search, which is based on random size cropping to predict the ROI, Faster R-CNN employs RPN [32]. RPN is much faster than the selective search on extracting candidate regions, because RPN reduces the number of candidates [31]. The detection results by selective search and RPN can be shown in Figure 11. As Figure 12 shows, the RPN generates much fewer proposal regions, with more accurate locations. Moreover, the ARE of Faster R-CNN, 0.24 m, is lower than the other, as the detection function contributes to the ranging performance. Thus, the model applying Faster R-CNN with 500 × 500 input has the best performance in the simulation experiment.

(2) Detection Performances in Different Visibility Conditions

**Figure 13:**
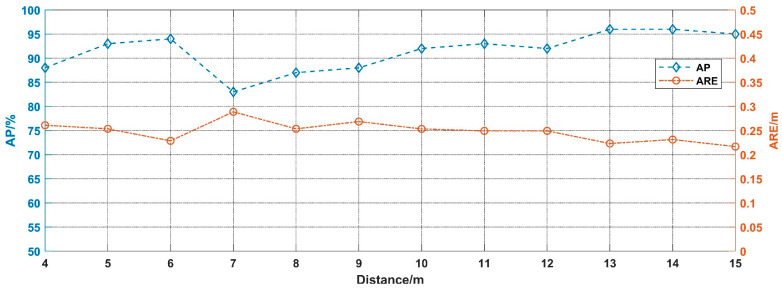
The performance in different visibility conditions.

The proposed method is evaluated by simulated signals of different visibility conditions ranging from 1 m to 15 m. According to the results, in conditions with visibility of 1 m, 2 m, and 3 m, the algorithm did not recognize the presence of target echo signal, which was in line with the fact that we have not annotated the ground truth in those spectrogram images. The remaining results are shown in Figure 13.

As can be seen in Figure 13, in conditions that visibility above 7 m, the AP decreases gradually, since the impact of noise increase with smoke concentration. It is worth noting that the AP of 6 m visibility is significantly improved compared with that of 7 m visibility. The possible reason is that the 2-D spectrogram images of 6 m visibility are measured from 13 m to 3 m, which are much closer, compared with the 7 m visibility chart, which measured from 16 m to 6 m. In addition, ARE increases slightly as visibility decreases, which indicates that the detection performance has some positive impact on the ranging function.

**Figure 14:**
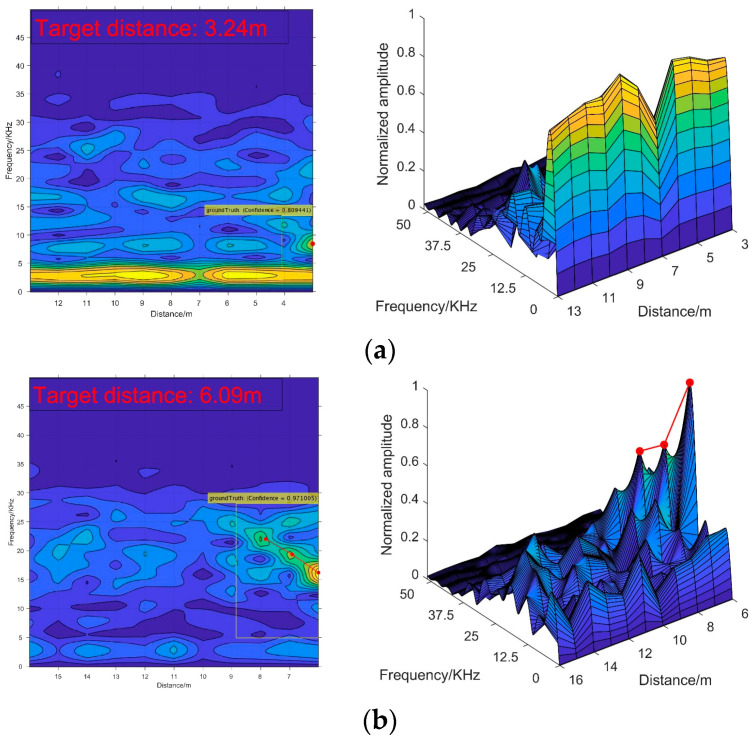
The detection performances in different visibility conditions: (**a**) results in a 4 m visibility condition; (**b**) results in a 14 m visibility condition.

Figure 14 shows detection results by utilizing the trained model on the test samples. It is clear that when the visibility is relatively high, the target echo signal has a strong intensity and is relatively significant in the 2-D spectrogram images, making it easy for recognition. As the visibility drops, the recognition becomes difficult, because the characteristics of target echo signal are not dramatic.

### 3.2. Experimental Results

#### 3.2.1. Experimental and Training Settings

**Figure 15:**
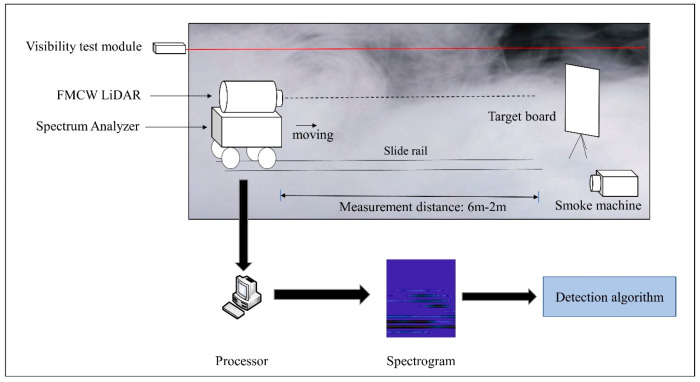
The experimental process in DVE.

In order to verify the method, a FMCW Lidar was applied to collect echo signal in DVE. Figure 15 demonstrates the experimental process. This experiment was conducted in a 7 m × 1.5 m × 2 m site, which was constructed using black flannel with a reflection coefficient of 0.05. The trolley carrying the Lidar and spectrum analyzer is placed 6 m away from the target board. At first, the smoke generator sprays smoke into the test site, the visibility test module measures the smoke concentration when the visibility is stable. Next, the trolley is pushed towards the target board at a constant speed. In this process, the echo signal is recorded every fixed distance. After that, the echo signals are computed by FFT to form 2-D spectrogram images. Eventually, the 2-D spectrogram images are sent into the proposed algorithm.

The construction details of the 2-D spectrogram images are shown in Table 3. Due to the limitation of the experiment site, the measurement distance of the experimental process is shorter than that of the simulation process. After the spectrogram images are constructed, the algorithm was trained with the same parameter settings as before.

#### 3.2.2. Experimental Results

(1) Comparison with Non-Deep Learning Methods

We compared this method with other non-deep learning methods, including the wavelet transformation method (Wavelet), the EMD direct wavelet thresholding method (EMD-DT), the EMD interval thresholding method (EMD-IT), and the correlation-based EMD partial reconstruction method (EMD-correlation). Since the denoise methods of non-deep learning are all for 1-D signals, we only use ARE and time consuming as the evaluation standard in this section. In order to ensure the fairness of the experiment, the 1-D echo signal closest to the target collected in the multi-distance measurement process is used as the input signal of the non-deep learning method.

It can be seen from Table 4 that, among the non-deep learning methods, compared with the EMD based methods, the wavelet transformation has a faster calculation speed and higher accuracy. The possible reason is that all of these EMD based methods rely on thresholds to eliminate noise, but the threshold settings for these methods are very rough and do not have a good denoising effect on noise. The proposed deep learning recognition method has the highest accuracy, as we do not need to consider setting a threshold for noise, we recognize the target echo signal based on the frequency-distance relation derived from the course of approaching the target.

(2) Comparison with Other Deep Learning Algorithms

We evaluated our method alongside other deep learning algorithms, and the results from different algorithms are shown in Table 5.

As Table 5 shows, similar to the simulation results, YOLO cannot effectively identify the target echo signal. The performances of 500 × 500 inputs are better than those of 227 × 227 inputs. The AP of R-CNN and Fast R-CNN with 500 × 500 are, respectively, 80.6% and 89.1%, while the Faster R-CNN with 500 × 500 is 92.3%. The ARE of Faster R-CNN with 500 × 500, 0.14 m, is lower than that of R-CNN and Faster R-CNN. It indicates that the Faster R-CNN with 500 × 500 inputs have the best detection performance, which is consistent with previous simulation conclusion. It should be noted that the experimental detection performance is better than that of simulation result. It is probably because the experiment process has a closer measurement distance compared with the simulation process.

(3) Detection performances in different visibility conditions

**Figure 16:**
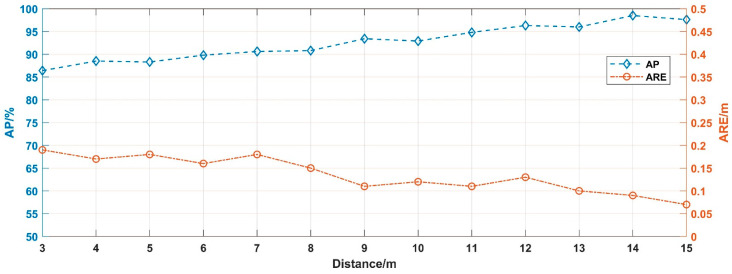
Detection results in different visibility.

We evaluated the detection performances of Faster R-CNN with 500 × 500 input in different visibility conditions ranging from 1 m to 15 m. The results are shown in Figure 16. Nevertheless, in conditions with visibility of 1 m and 2 m, the algorithm did not recognize the presence of the target echo signal, which was in line with the fact that we have not annotated the ground truth in those images. In this section, the algorithm can recognize the target echo signal at a visibility of 3 m, but in the simulation result it cannot be recognized, the possible reason being that the measurement distance (from 6 m to 2 m) in the experimental process is closer than the simulation process (from 13 m to 3 m).

**Figure 17:**
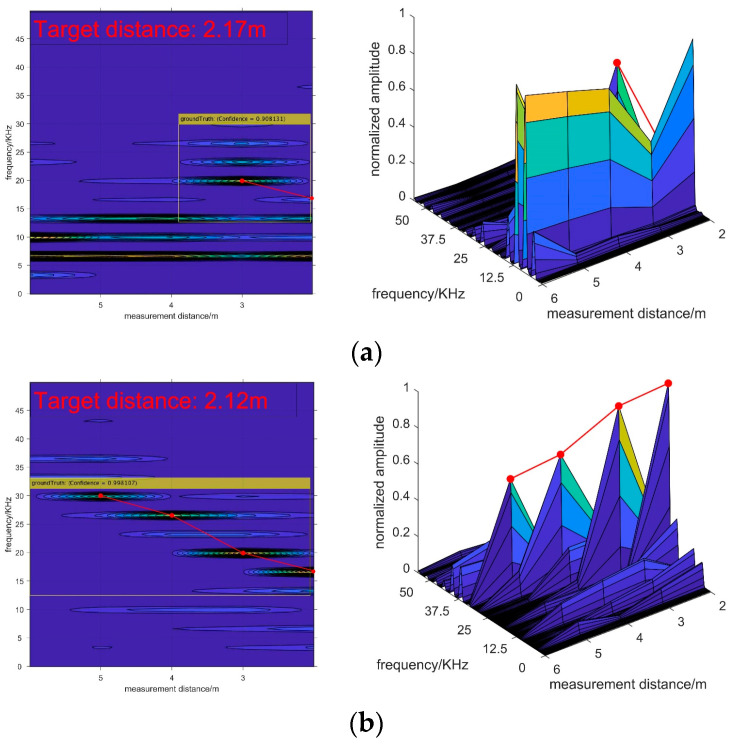
The detection performances in different visibility conditions: (**a**) results in a 4 m visibility condition; (**b**) results in a 14 m visibility condition.

As presented in Figure 16, as the measurement distance is settled, the AP decreases gradually with increasing smoke concentration, while the ARE slightly increases. The results of the experiment prove the conclusions of the previous section. In addition, the detection performances of the test samples are shown in Figure 17.

However, as Figure 14 and Figure 17 demonstrate, there are some differences between the simulation and experiment signals that need to be explained. As the target echo signals appear as a continuous ridge shape in the simulation images, while they look like independent mountain peaks in the experimental images. The reason is that the frequency resolution of the experimental signal is higher than the simulated signal. Therefore, the adjacent spectral peaks in the measured data are separated, while they are continuous in the simulated data. These occasions lead to wide main lobe width of the simulation spectrums while the main lobe widths of the experimental spectrums are narrow. In conclusion, the characteristics of target echo signals in the 2-D spectrogram images obtained from the experiment still obey the recognition criterion.

## 4. Conclusions

In this paper, we have proposed a novel obscurant penetrating detection method for Lidar in DVE. As automatic driving is rapidly developing, our proposed method is applicable to mobile application scenarios. The proposed multi-distance measurement method can collect multiple 1-D echo signals during movement. An effective target echo signal recognition method is also presented, as the 2-D spectrogram images contain dynamic characteristics of 1-D echo signal sequence in the process of approaching the target. Our method requires no denoising and suppression functions to process backscattering signal. Furthermore, we have proposed a recognition algorithm based on deep learning to recognize the target echo signal. The simulation and experimental results have verified that the proposed method can be well trained and utilized for Lidar detection in DVE. In the future, we will transplant the algorithm to Field Programmable Gate Array (FPGA) and consider practical application problems.

## Figures and Tables

**Table 1:** Construction details about 2-D spectrogram images.

Visibility/m	Measurement Distance	Measurement Times	Annotation Situation
1−3	13 m to 3 m	11	No
4−6			Yes
7−15	16 m to 6 m		

**Table 2:** The results of the different algorithms.

Methods	Resolution	AP (%)	ARE (m)	Time(s)
YOLO	227 × 227	0	∞	0.02
	500 × 500	0	∞	0.08
R-CNN	227 × 227	57.0	2.75	5.63
	500 × 500	75.1	1.32	53.34
Fast R-CNN	227 × 227	75.9	2.53	1.24
	500 × 500	86.3	1.34	4.42
Faster R-CNN	227 × 227	85.0	1.91	0.49
	500 × 500	91.4	0.24	0.86

**Table 3:** Construction details of the spectrogram images.

Visibility/m	Measurement Distance	Measurement Times	Annotation Situation
1−2	6 m to 2 m	5	No
3−15			Yes

**Table 4:** Comparison with non-deep learning methods.

Methods	Wavelet	EMD-DT	EMD-IT	EMD-Correlation	Our Method
**ARE(m)**	0.32	0.48	0.42	0.45	0.14
**Time(s)**	0.82	5.27	4.86	4.42	0.75

**Table 5:** The results of the different algorithms.

Algorithms	Resolution	AP (%)	ARE (m)	Time(s)
YOLO	227 × 227	0	∞	0.04
	500 × 500	0	∞	0.11
R-CNN	227 × 227	62.9	1.46	6.72
	500 × 500	80.6	0.81	46.65
Fast R-CNN	227 × 227	78.3	1.08	0.95
	500 × 500	89.1	0.34	4.48
Faster R-CNN	227 × 227	82.5	0.84	0.34
	500 × 500	92.3	0.14	0.75
